# Transthoracic Echocardiogram Guided Hemodynamic Management to Maintain Cerebral Perfusion in an Extracranial–Intracranial Bypass Case: Case Report

**DOI:** 10.1055/a-2650-6679

**Published:** 2025-07-22

**Authors:** Beddome C. Allen, Chesney S. Oravec, Stacey Q. Wolfe, Saraschandra Vallabhajosyula, Sahil Kapoor, Sai Varun Bethina, Aarti Sarwal

**Affiliations:** 1 Department of Neurology UH Cleveland Medical Center, Cleveland, Ohio, United States; 2 Department of Neurological Surgery, Atrium Health Wake Forest Baptist Medical Center, Winston-Salem, North Carolina, United States; 3 Department of Medicine, Section of Cardiovascular Medicine, Wake Forest University School of Medicine, Winston-Salem, North Carolina, United States; 4 Department of Psychiatry, Baptist Health-University of Arkansas for Medical Sciences, Little Rock, Arkansas, United States; 5 Department of Neurology, Virginia Commonwealth University, Richmond, Virginia, United States

**Keywords:** echocardiography, carotid stenosis, hypertrophic cardiomyopathy, transient ischemic attack, cerebrovascular circulation, cerebral revascularization

## Abstract

A 70-year-old man with transient ischemic attacks (TIAs) due to left internal carotid artery (ICA) occlusion underwent external carotid to ICA bypass which occluded postoperatively. He developed aphasia which resolved with induced hypertension optimized by using serial transthoracic echocardiography in the setting of left ventricle outflow tract obstruction.

## Background

Medical management for intracranial or extracranial large artery atherosclerotic disease includes blood pressure control, cholesterol lowering agents, lifestyle modifications, glycemic control, and use of antithrombotic agents. 1 2 Patients with flow-limiting carotid stenosis may be candidates for endarterectomy or stenting to restore flow, 3 4 but chronically occluded carotid arteries are typically not amenable to these therapies. 5 6 7 8 Most patients develop collateral circuits through channels like the ophthalmic artery, anterior communicating artery, or leptomeningeal arteries among other vessels to maintain cerebral perfusion to the brain. Patients with flow-limiting carotid occlusion and inadequate collateralization at normal blood pressures may additionally require induced hypertension to maintain adequate cerebral perfusion pressure (CPP) to prevent cerebral ischemia. 9 10

Revascularization with direct or indirect external carotid artery to internal carotid artery (EC-IC) bypass is commonly done in Moya-Moya disease but for carotid occlusion related to atherosclerotic disease, 11 it is a last resort reserved for patients who have failed maximal medical management. 4 12 13 Patients with flow-limiting carotid occlusion with recurrent strokes who have failed maximum medical management are sometimes managed with induced hypertension to help engage collateralization or referred for EC-IC bypass if there are concerns for adverse effects related to hypertension. Postoperative care of patients undergoing bypass is challenging and may be complicated by perioperative stroke due to direct bypass occlusion or hemodynamic fragility. 14 Postoperative stroke rates can be as high as 31%. 15 Studies assessing a comprehensive cardiac assessment have been lacking. Many of these patients with cerebrovascular risk factors have long-standing hypertension, which may be accompanied by occult left ventricular outflow tract (LVOT) obstructive physiology, similar to hypertrophic obstructive cardiomyopathy (HOCM). In these cases, inducing hypertension can counterproductively reduce cardiac output, which decreases CPP and may contribute to postoperative stroke.

We present the case of a patient with ICA occlusion who underwent EC-IC bypass with subsequent bypass occlusion who experienced stroke symptoms at normal blood pressures necessitating induced hypertension but again experienced neurologic deterioration despite induced hypertension. He was found to have cardiac physiology concerning for LVOT obstruction with dynamic underfilling related to vasopressor use causing impaired cardiac output at high blood pressures. This necessitated blood pressure goals in a tight range with concern for flow-limiting stenosis-related cerebral perfusion deficit at lower blood pressures and impaired cardiac output from LVOT obstruction physiology causing impaired cerebral perfusion at higher blood pressures. This case highlights the need to include comprehensive cardiopulmonary assessments in cases requiring vasopressors to augment blood pressure.

## History of Presentation

A 70-year-old male with paroxysmal atrial fibrillation, encephalitis (remote history), transcatheter aortic valve replacement (TAVR) 2 years prior, and prior myectomy for HOCM complicated by brief pulseless electrical activity requiring cardiopulmonary resuscitation necessitating a loop recorder placement was maintained on ASA 81 mg daily and presented with episodes of right-side hemiparesis and dysarthria occurring one to two times per week over 2 months. The patient provided informed consent to have images and health information published. The frequency of symptoms increased to four to five transient ischemic attacks (TIAs) daily despite compliance with aspirin and aggressive cholesterol and glycemic control.

## Investigations

**Fig. 1:**
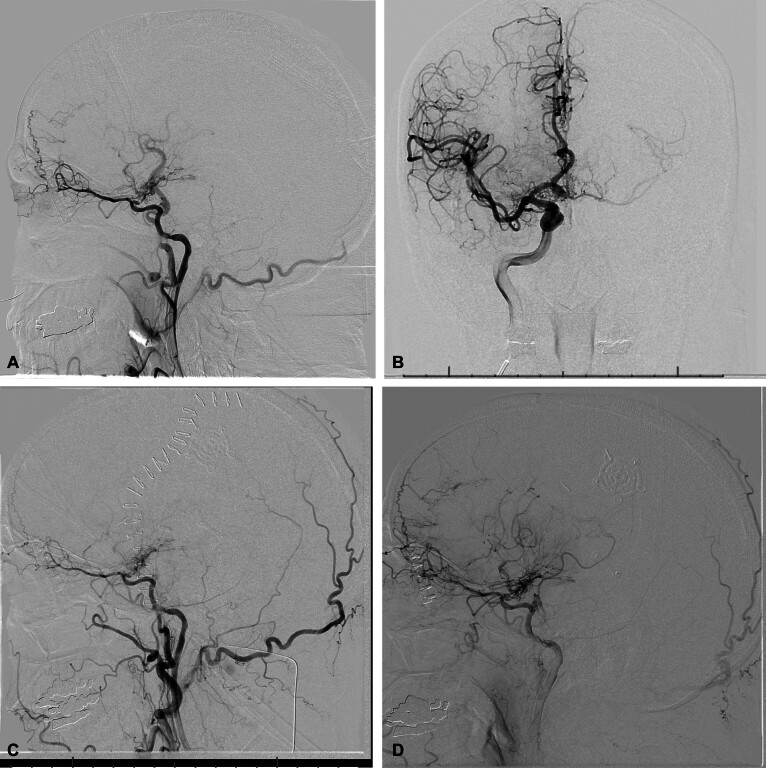
( **A** ) Preoperative digital subtraction angiography demonstrating left internal carotid artery (ICA) occlusion. ( **B** ) Preoperative digital subtraction angiography of right ICA demonstrating filling of the distal left anterior cerebral artery (ACA) and left middle cerebral artery (MCA) territories primarily via pial collaterals and anterior communicating artery supply from the right ICA. ( **C** ) Postoperative digital subtraction angiography demonstrating occluded superficial temporal artery–middle cerebral artery (STA-MCA) bypass with patent collateral vessels on day 1 after surgery. ( **D** ) Postoperative digital subtraction angiography demonstrating ongoing dural–pial collateral vessels on day 180 after surgery.

Computed tomography angiogram (CTA) of head and neck demonstrated atresia of the left cervical ICA beginning distal to the carotid bifurcation with occlusion of the ICA terminus. After evaluation by vascular neurosurgery in collaboration with the patient's cardiologist, the patient was managed with dual antiplatelet therapy, aspirin 325 mg and clopidogrel 75 mg daily. His home antihypertensive regimen of metoprolol and torsemide was stopped to promote permissive hypertension and facilitate left-sided cerebral blood flow by engaging collateralization. Diagnostic cerebral angiogram (DSA) revealed occlusion of the left supraclinoid ICA and anterior cerebral artery ( Fig. 1A ). Filling of the distal left anterior cerebral artery and left middle cerebral artery territories occurred primarily through pial collaterals and anterior communicating artery supply from the right ICA ( Fig. 1B ). A robust left superior temporal artery was noted.

## Management

Due to no resolution of daily TIAs, moderate hypertension was induced with a goal systolic blood pressure (SBP) of >140 mmHg with midodrine 5 mg twice daily. Careful blood pressure monitoring by family at home revealed a reproducible association of TIA episodes with SBPs of 120 to 130 mmHg that resolved or did not occur when SBPs consistently stayed above 160 mmHg. This raised concern for insufficient CPP; therefore, midodrine was titrated to 10 mg three times daily (TID) to maintain SBP greater than 140 to 160 mmHg reducing TIA frequency significantly. Vasomotor reactivity via transcranial Doppler and perfusion imaging were not pursued as the patient's transient right hemiparesis and dysarthria were clinically related to left hemisphere hypoperfusion in the setting of low SBP with known left ICA occlusion.

With ongoing concern through multidisciplinary consultations that maximum medical management was unsuccessful, and that prolonged hypertension would have adverse effects, the decision was made to pursue a direct left superficial temporal artery–middle cerebral artery (STA-MCA) bypass and encephaloduroarteriosynangiosis (EDAS). EDAS was considered to enhance postoperative vascular collateral development with the ICA occlusion suspected to be secondary to prior encephalitis, not secondary to atherosclerosis. The STA-MCA bypass and EDAS were performed without technical issues. The cross-clamp time of the STA and recipient MCA was 37 minutes. An indocyanine green videoangiography was not performed but intraoperative Doppler demonstrated patency of the STA and proximal/distal MCA recipient with reversed flow following the bypass. The postoperative course was complicated by several episodes of sinus pauses, with the longest lasting 4.5 seconds, associated with transfer from the operating room table to the transport stretcher ascribed to Valsalva maneuver during transfer. No transient hemiparesis or dysarthria occurred. DSA performed on postoperative day 1 to follow up on patency of the graft demonstrated occlusion of the left STA-MCA bypass with ongoing collateral filling ( Fig. 1C ). The patient remained neurologically intact and was monitored in the neurosciences intensive care unit with target SBP goal of 160 to 180 mmHg on oral midodrine 10 mg three times a day and norepinephrine drip. Dual antiplatelet therapy regimen was continued, and enoxaparin 30 mg twice daily was added for deep vein thrombosis prophylaxis as per standard institutional protocol.

**Fig. 2:**
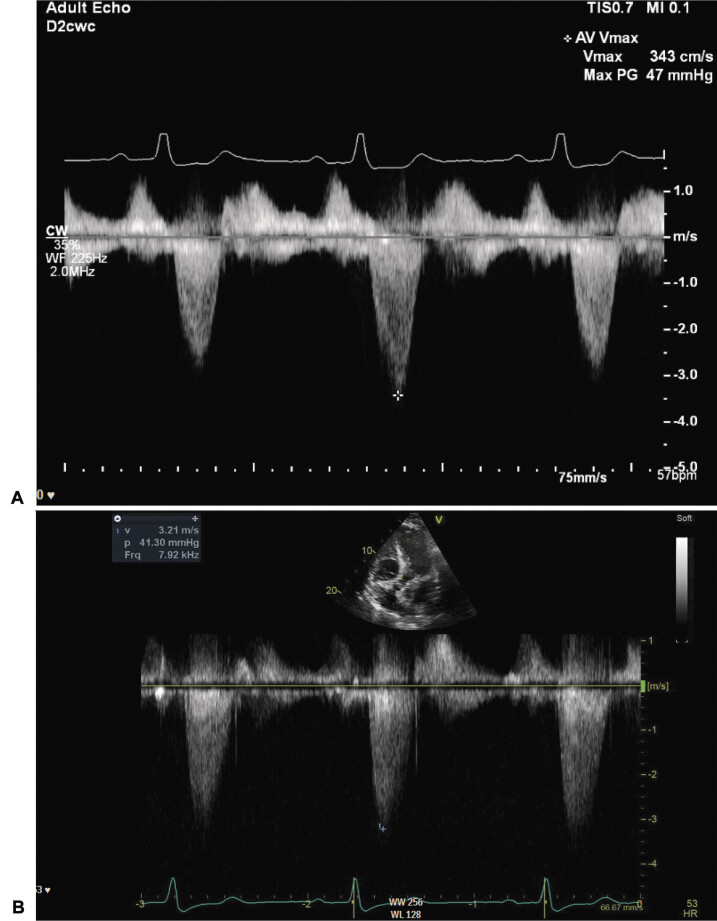
( **A** ) Velocity waveform across aortic valve with goal systolic blood pressure of 160 to 180 mmHg measured by transthoracic echocardiography (TTE). ( **B** ) Velocity waveform across arotic valve with goal systolic blood pressure 160 mmHg by TTE.

On postoperative day 3, the patient began experiencing intermittent episodes of hypotension with SBP of 90 mmHg while at rest despite escalating doses of vasopressors and ensuring euvolemia based on net fluid balance. Indwelling loop recorder interrogation revealed several episodes of sinus pauses and complete heart block intraoperatively and immediately postoperatively. No complete heart block occurred after postoperative day 1 and none coincided with neurological deterioration or hypotension. Due to known prior HOCM and TAVR, echocardiography was obtained to further evaluate cardiac physiology as a contributor to hypotension. Transthoracic echocardiography demonstrated normal left ventricular systolic function (ejection fraction 55–60%), asymmetrical septal hypertrophy, flow acceleration in the LVOT, and no systolic anterior motion of anterior mitral leaflet. There was evidence of a fixed LVOT obstruction with an elevated peak transaortic valve gradient of 47 mmHg concerning for increase in prosthetic aortic valve gradients with dynamic left ventricle (LV) underfilling ( Fig. 2A ).

With consultation among neurocritical care, cardiac electrophysiology, and vascular neurosurgery, the SBP goal was modified to about 160 mmHg in order to decrease LV afterload while maintaining cerebral perfusion. With this SBP goal, the patient experienced no subsequent hypotensive episodes or episodes of neurologic deterioration. Repeat echocardiogram demonstrated decrease in the peak transaortic gradients to 40 mmHg ( Fig. 2B ). Norepinephrine was weaned due to concerns of worsening LV underfilling related to LV hypercontractility, and the patient was transitioned to midodrine three times a day (with dosing regimen of 5 mg/2.5 mg/5 mg daily) while maintaining ASA 325 mg and clopidogrel 75 mg daily. Since the patient's neurologic examination was stable with new BP goals and because he tolerated the transition to PO midodrine, the risk of surgery outweighed the benefit; therefore, surgical revision was deferred.

The patient continued to follow his blood pressures closely with attempts to maintain SBP at 140 to 160 mmHg even after hospital discharge. He had few TIAs subsequently that completely resolved by 3 months. No postoperative MRI of the brain was obtained due to implanted loop recorder incompatibility. Midodrine was weaned off at 6 months without any subsequent symptoms. Follow-up DSA at 6 months ( Fig. 1D ) demonstrated dural–pial MCA vascular collaterals in the area of the motor strip, demonstrating the success of the indirect EDAS despite the occluded STA-MCA bypass. Transcranial Doppler vasoreactivity at the 6-month appointment was low normal at 29% indicating adequate vasodilatory reserve with a reduced stroke risk. ASA was stopped and clopidogrel monotherapy was continued after confirming clopidogrel response with platelet light aggregation testing.

## Discussion

LV hypercontractility from vasopressor use in the presence of fixed LVOT obstruction can contribute to decreased cerebral perfusion and may be an unrecognized factor in postoperative strokes in patients requiring vasopressors for induced HTN to increase CPP, such as patients with a failed STA-MCA bypass. 16 17 Dynamic LVOT obstruction has been reported in patients with aortic valve replacement for aortic stenosis and hypertensive hypertrophy as well as in structurally normal hearts with relative hypovolemia treated with catecholamines. 18 19 In this challenging case of a patient with an occluded left STA-MCA bypass and underlying cardiac pathology, we demonstrate the utility of comprehensive cardiac assessment in guiding the hemodynamic management of a patient needing systemic blood pressure augmentation to optimize cerebral perfusion. Although this patient had known HOCM pathology with prior myectomy, he demonstrated dynamic LV underfilling with a fixed aortic valve obstruction that worsened with initiation of vasopressors augmenting LV contractility. Transthoracic echocardiogram enabled the appropriate selection of target blood pressure goals by maintaining some degree of induced hypertension with midodrine in this patient.

## Conclusion

In ICU patients requiring vasopressors, especially for induced hypertension, who are experiencing transient neurologic deterioration despite hypertension, it may be useful to evaluate cardiac structure and function with serial echocardiography to ensure no adverse impact of vasopressors on cardiac output. In this case, echocardiography assisted the identification of a safe induced hypertension goal range in a patient with dynamic LV underfilling caused by LV hypercontractility in the presence of fixed LVOT obstruction who also needed some degree of high blood pressure due to occluded ICA and a recently occluded STA-MCA bypass.

## Learning Objectives

Case: A patient who presented with TIAs and underwent EC-IC bypass.

1. To understand the indications for EC-IC bypass.
2. To understand one of the challenges of maintaining optimal cerebral perfusion in patients with LVOT obstruction.
